# The Relationship between Types of Attention and Auditory Processing Skills: Reconsidering Auditory Processing Disorder Diagnosis

**DOI:** 10.3389/fpsyg.2018.00034

**Published:** 2018-01-30

**Authors:** Georgios Stavrinos, Vassiliki-Maria Iliadou, Lindsey Edwards, Tony Sirimanna, Doris-Eva Bamiou

**Affiliations:** ^1^Ear Institute, Faculty of Brain Sciences, University College London, London, United Kingdom; ^2^Neuroscience Division, Aristotle University of Thessaloniki, Thessaloniki, Greece; ^3^Psychological Services, Great Ormond Street Hospital, London, United Kingdom; ^4^Department of Paediatric Audiology, Great Ormond Street Hospital, London, United Kingdom; ^5^Department of Neuro-Otology, National Hospital for Neurology and Neurosurgery, London, United Kingdom; ^6^Biomedical Research Centre, National Institute for Health Research, London, United Kingdom

**Keywords:** auditory processing disorder, attention deficits, diagnostic criteria, attention, audiology, paediatrics

## Abstract

Measures of attention have been found to correlate with specific auditory processing tests in samples of children suspected of Auditory Processing Disorder (APD), but these relationships have not been adequately investigated. Despite evidence linking auditory attention and deficits/symptoms of APD, measures of attention are not routinely used in APD diagnostic protocols. The aim of the study was to examine the relationship between auditory and visual attention tests and auditory processing tests in children with APD and to assess whether a proposed diagnostic protocol for APD, including measures of attention, could provide useful information for APD management. A pilot study including 27 children, aged 7–11 years, referred for APD assessment was conducted. The validated test of everyday attention for children, with visual and auditory attention tasks, the listening in spatialized noise sentences test, the children's communication checklist questionnaire and tests from a standard APD diagnostic test battery were administered. Pearson's partial correlation analysis examining the relationship between these tests and Cochrane's Q test analysis comparing proportions of diagnosis under each proposed battery were conducted. Divided auditory and divided auditory-visual attention strongly correlated with the dichotic digits test, *r* = 0.68, *p* < 0.05, and *r* = 0.76, *p* = 0.01, respectively, in a sample of 20 children with APD diagnosis. The standard APD battery identified a larger proportion of participants as having APD, than an attention battery identified as having Attention Deficits (ADs). The proposed APD battery excluding AD cases did not have a significantly different diagnosis proportion than the standard APD battery. Finally, the newly proposed diagnostic battery, identifying an inattentive subtype of APD, identified five children who would have otherwise been considered not having ADs. The findings show that a subgroup of children with APD demonstrates underlying sustained and divided attention deficits. Attention deficits in children with APD appear to be centred around the auditory modality but further examination of types of attention in both modalities is required. Revising diagnostic criteria to incorporate attention tests and the inattentive type of APD in the test battery, provides additional useful data to clinicians to ensure careful interpretation of APD assessments.

## Introduction

Auditory Processing Disorder (APD) is characterised by normal peripheral hearing, but abnormal processing of auditory information within the central auditory nervous system (Martin and Keith, 2009) and by deficits in sound-in-noise discrimination (Dawes and Bishop, 2009; Lagacé et al., 2011; Keith and Purdy, 2014). Among other symptoms in children with APD, poor auditory attention span and distractibility are commonly reported (American Speech-Language-Hearing Association, 2005a; American Academy of Audiology, 2010). The influence of cognitive top-down functions on Auditory Processing (AP) tests is a point of scientific debate between the British Society of Audiology (British Society of Audiology, 2011a)^1^ and the American Speech-Language Hearing Association (American Speech-Language-Hearing Association, 2005b)^2^ The British Society of Audiology argues that cognitive factors, such as attention and memory, influence AP (British Society of Audiology, 2011a), whereas the American Speech-Language Hearing Association states that deficits in the auditory pathway alone should define APD (American Speech-Language-Hearing Association, 2005b). This discussion directly impacts selection of diagnostic tests and APD diagnosis.

Attention is being defined as an individual's selection from a multitude of available sensory information (Hahn et al., 2008), while these selected stimuli are perceived and then processed (Broadbent, 1958). Sustained attention is the vigilant focus on stimuli and is considered a basic function that determines selective and divided attention (Sarter et al., 2001). Selective attention is the process of allocating resources on specific input, whereas divided attention is the process of resource allocation between different stimuli by rapidly shifting or splitting focus (Parasuraman, 1998). Despite different definitions for types of attention, engagement of brain activity is merely qualitatively different without specific neural circuits responsive to them (Hahn et al., 2008).

Previous studies on children with suspected APD found associations between Sustained Auditory Attention (Sus-AA) and AP tests (Sharma et al., 2009; Gyldenkærne et al., 2014; Tomlin et al., 2015; Cameron et al., 2016), while another found worse Divided Auditory Attention (Div-AA) test scores in children with dichotic listening deficits (and suspected of but not diagnosed with APD) compared to normal children (Martin et al., 2007). A study demonstrated that a Divided Auditory-Visual Attention (Div-AVA) task did not predict APD (Lam and Sanchez, 2007). Nonetheless, the association between divided attention tasks and individual AP tests from an APD diagnostic battery has not been examined. Divided auditory attention and the dichotic digits test both require divided listening, while there are indications that both Div-AA and dichotic listening are using interhemispheric transfer and that deficits in these two functions might both be involving the corpus callosum (Hutchinson et al., 2008; Westerhausen and Hugdahl, 2008; Musiek and Weihing, 2011; van der Knaap and van der Ham, 2011). Examining the relationship between Div-AA and dichotic listening could help determine the nature of dichotic deficits in children with APD. Regarding Div-AVA, it is less clear how that could be related to AP deficits, but it is worth examining this type of attention in children with APD. The auditory and visual modality in divided attention tasks make use of attentional resources from a shared pool of resources (Wahn and König, 2017) and this function could be affected by a compromised auditory modality in children with APD. Some studies report poor scores in sustained visual attention tests in children suspected of APD (Sharma et al., 2009; Gyldenkærne et al., 2014) and correlations between sustained visual attention and some AP tests (Cameron et al., 2016), but the relationship between APD and other types of visual attention, such as Selective Visual Attention (Sel-VA), has not been investigated. The nature of Attention Deficits (ADs) in children with APD is not very well laid out thus far. Investigating further visual attention measures, such as Sel-VA, is important as possible deficits in visual attention measures could point to more global attentional deficits (visual and auditory) in children diagnosed from the APD battery as having APD.

Standard procedures for diagnosing APD usually include, audiological tests, auditory brainstem tests (Bamiou et al., 2006) and AP tests (American Speech-Language-Hearing Association, 2005a; Cowan et al., 2009; Martin and Keith, 2009; Ferguson et al., 2011). These tests may be influenced by cognitive factors, such as sustained auditory attention (Sharma et al., 2009; Dillon et al., 2012; Gyldenkærne et al., 2014; Tomlin et al., 2015). Cognitive skills measured through the auditory modality, though, may also be affected by the mode of test administration. These associations between attention and AP tests suggest that APD diagnostic criteria need to be reconsidered, as they do not currently routinely include cognitive diagnostic measures. A study comparing nine different but widely used sets of criteria for APD diagnosis, on a sample of 150 Australian children, found substantial differences in diagnosis rates between these sets of criteria (Wilson and Arnott, 2013). However, measures of auditory attention were not incorporated in any of the criteria, despite indications that sustained auditory attention may interact with AP skills (Sharma et al., 2009; Gyldenkærne et al., 2014; Tomlin et al., 2015; Cameron et al., 2016).

Drawing from these findings, our pilot study aims to examine the relationships between different types of attention and AP abilities in order to improve the management and treatment strategies for children with AP difficulties by better identifying their needs through newly proposed diagnostic procedures. The study will first examine the associations between types of attention and AP tests by analysing data from children that met specified APD diagnostic criteria, an analysis that other studies have not examined. Previous research has only looked at these relationships in a mixed sample of children suspected of and diagnosed with APD and mainly focused on Sus-AA and AP skills, while other types of attention (such as divided and visual) have not been sufficiently investigated. It is hypothesised that in addition to Sus-AA correlating with specific AP measures, as previously shown (Sharma et al., 2009; Gyldenkærne et al., 2014; Tomlin et al., 2015; Cameron et al., 2016), divided measures of attention will also correlate with AP tasks that require binaural integration, while Sel-VA will not correlate with AP measures. Next, the study will compare the yield of the APD standard diagnostic battery with the yield of a battery looking for Attention Deficits (ADs) and with the yield of a battery testing for APD but excluding AD cases. It is hypothesised that the standard APD battery will have a greater yield than the AD battery; this was expected as children in the initial sample of suspected APD were referred for APD assessment but also previous studies (even though not statistically testing for it as it was not in their aims) in their suspected APD samples recorded larger proportions of APD diagnosis (72%) over auditory plus visual attention deficits (41%) (Sharma et al., 2009) and 49% over 15%, respectively, in another study (Gyldenkærne et al., 2014). Lastly, the study will discuss the diagnostic battery required to identify APD children with predominantly inattentive behaviours, termed here as “inattentive subtype of APD” (i-APD). This term was adapted from the predominantly inattentive subtype of children with Attention Deficit Hyperactivity Disorder (ADHD) (Chermak et al., 2002).

## Materials and methods

### Participants

Initially, 85 invitation letters were sent to families of children with parental reports of Speech-in-Noise (SiN) difficulties and with referrals by audiologists for APD assessment at Great Ormond Street Hospital in London on suspicion of AP deficits. Thirty replies were received and an inclusion criterion of at least a *T* score of 85 on a non-verbal Intelligence Quotient (IQ) test was set. This excluded three children who were below that threshold. Thus, the study sample comprised 27 UK children (9 girls and 18 boys) suspected of APD, aged 7–11 years (mean age 9 years 7 months). All children had English as first language and three were bilingual. None of the children previously used a Frequency Modulation (FM) system or auditory training. None met diagnostic criteria for ADHD. All children and parents signed assent and consent forms, respectively. The Bloomsbury Research Ethics Committee approved the study.

### Tests and procedure

All children were tested for APD following the standard APD diagnostic procedure. First, pure tone audiogram and tympanogram/ acoustic reflexes were used to confirm normal peripheral hearing. Children completed AP tests at Great Ormond Street Hospital as per the clinic's APD protocol. The AP tests comprised the Dichotic Digits Test (DDT), Gaps-in-Noise (GiN), Frequency Pattern Test (FPT), and SCAN-3 Auditory Figure Ground (AFG) +8 dB and 0 dB. The DDT presents 20 stimuli (with 2 digits per ear, from 1 to 10 excluding 7) and the child needs to repeat back the numbers from both ears (Guenette, 2006). The GiN test presents 6-s broadband noises in each ear, containing 0–3 silent gaps and the child needs to detect these gaps (Paulovicks, 2008). The FPT has low (880 Hz) and high (1,122 Hz) tones presented in successive patterns of triplets (30 patterns in each ear), with a duration of 150 ms, an intertonal interval of 200 ms and the child is asked to detect and verbally repeat these patterns (Musiek and Pinheiro, 1987; Musiek, 2002). The SCAN-3 AFG +8 dB condition has the target voice over the background babble noise at 8 dB greater intensity, while for the SCAN-3 0 dB target and background noise have the same intensity (Keith, 2009).

Three additional tests were administered at University College London Ear Institute on a separate date. These were the Wechsler Non-Verbal (WNV) Scale of Ability, the Test of Everyday Attention for Children (TEACh) and the Listening in Spatialized Noise-Sentences (LiSN-S) test (Manly et al., 1999; Wechsler and Naglieri, 2006; Cameron and Dillon, 2011). The IQ test was chosen to be non-verbal in order to measure cognitive ability without influences by possible language problems. The two sub-tests administered from WNV were Matrices (measuring general ability and perceptual reasoning) and Spatial Span (measuring general ability and visual working memory) and combined *T* scores from both these tests comprise a composite score which calculates overall IQ score (Wechsler and Naglieri, 2006). The four sub-tests used from TEACh were Sky Search, Score, Sky Search Dual Task and Score Dual Task measuring respectively Sel-VA, Sus-AA, Div-AVA, and Div-AA (Manly et al., 1999). In the Sel-VA task, children found visual targets among distractors, in the Sus-AA test they performed a simple but uninteresting task (counting sounds with long gaps in between), in the Div-AVA test they did the same task as in the first test plus a similar one from the second test (with fixed and short gaps in between sounds instead) and in the Div-AA task children counted sounds, as before, while they simultaneously listened for a target word in a news broadcast (Manly et al., 1999).

In both the TEACh and LiSN-S tests the child sat opposite the tester. In TEACh, a Phillips CD Player (Soundmachine AZ105S) connected to two ALTEC LANSING speakers was used with the sound level measured at ear level from the child's seat at 60 dB sound pressure level with a calibrated Casella CEL-450 sound level meter. The LiSN-S test ran on a ProBook HP laptop via the Phonak soundcard and Sennheiser HD125 headphones, which kept competing speech (either coming from the side or front) at a constant level of 55 dB sound pressure level and frontal target sentences at an initial 62 dB sound pressure level, then adjusted 2 dB up or down if at least 50% of the sentence words were incorrect or correct, respectively (Cameron and Dillon, 2008). Test order was randomised for each child. Breaks were given whenever needed and the total duration for WNV, TEACh, and LiSN-S tests was approximately 1 h and 30 min.

Finally, the CCC-2 questionnaire was given to parents to complete. The questionnaire is a tool that identifies presentations or symptoms comorbid with APD, such as Specific Language Impairment (SLI) (Ferguson et al., 2011; Miller and Wagstaff, 2011). The diagnoses of Autism Spectrum Disorder (ASD), Pragmatic Language Impairment (PLI) or SLI from the CCC-2 were based on the following guidelines; ASD diagnosis if sub-scales “I” and “J” were below the 6th percentile and the General Communication Composite score below 55, PLI diagnosis if the Social Interaction Deviance Composite was below 0 and the General Communication Composite score below 55, and SLI diagnosis if Social Interaction Deviance Composite score was between 0 and 9 and General Communication Composite score below 55 (Bishop, 2003).

### Diagnostic criteria

Diagnostic APD Battery (following the standard APD diagnostic protocol) identified children as having APD when at least two of the AP test scores were 2 Standard Deviations (SDs) below the mean or when only one test was 3 SDs below the mean (American Speech-Language-Hearing Association, 2005a)^3^. If none of these conditions were met but disordered score patterns were recorded on LiSN-S (2 SDs below the mean in Spatial Advantage and High-cue or Total Advantage conditions), then the child was diagnosed with Spatial Processing Disorder (SPD), a subtype of APD (Cameron and Dillon, 2011). Children with SPD face problems segregating frontal target speech when distracting sounds arrive from other directions (Cameron and Dillon, 2011). Children were categorised under each of the four batteries included in the study, as seen in Table 1. The first is the standard APD diagnostic battery described above, while the other three are part of the diagnostic procedure the study proposes. The i-APD battery identifies children with APD having only one additional failed attention test (below 2 SDs from the mean), thus screening for APD children with an inattentive APD subtype. If two or more attention tests are found disordered, then they are not considered i-APD, as these children might have comorbid ADs and APD, instead.

**Table 1:** The four batteries used in the study and their criteria for diagnosis and categorisation.

	**Criteria for diagnosis/categorisation**
APD Battery	At least two AP tests−2 SDs from the mean or one AP test−3 SDs from the mean or−2 SDs from the mean on the LiSN-S conditions of Spatial Advantage and High cue/Total Advantage.
AD Battery	At least two TEACh sub-test scores−2 SDs from the mean.
APD (without ADs) Battery	Diagnosed with APD under APD Battery but not identified with ADs under AD Battery.
i-APD Battery	Diagnosed with APD under APD Battery and only one TEACh sub-test score−2 SDs from the mean.

*AD, Attention Deficit; AP, Auditory Processing; APD, Auditory Processing Disorder; i-APD, inattentive subtype of APD; LiSN-S, Listening in Spatialized Noise–Sentences; SD, Standard Deviation; TEACh, Test of Everyday Attention for Children*.

### Statistical analyses

Since association of auditory processing skills could be different between normal and disordered children or between other groups with developmental disorders (Grube et al., 2014; Kuppen and Goswami, 2016), we examined associations in the subgroups of children who fulfil specific diagnostic criteria of APD in order to address the concern that extrapolating inferences from APD suspected to APD diagnosed children might be problematic (Iliadou et al., 2016). To examine the relationships between types of attention and AP tests, Pearson's partial correlation analysis was used. Gender and IQ scores were controlled in this model, despite conflicting findings of the latter's influence on auditory performance (Rosen et al., 2010), since the sample we used (APD diagnosed children only) has not been studied previously under these conditions. The FPT was removed from the analysis, since the correlation sample size comprised only 6 children, which makes it impossible to produce meaningful results. In the second part of the analysis, Cochran's Q test was used to compare the proportions from the three batteries (APD, AD, APD without ADs). This analysis looks at differences in proportions of identification between groups.

## Results

### Audiometry

All 27 children had normal hearing thresholds (below 20 dB for each frequency between 250 Hz and 8 KHz) (British Society of Audiology, 2012)^4^, normal middle-ear pressure (between−150 and +50 daPa), normal middle-ear admittance (between 0.3 and 1.6 cm^3^) and typical ear-canal volumes (between 0.4 and 1.0 cm^3^) (British Society of Audiology, 2013)^5^.

### Auditory processing and attention tests

Mean proportions of disordered scores in the attention and AP tests are summarised in Table 2. Children were classified as “Normal” when their scores were above−1 SD, “Borderline” when between−2 and−1 SDs and “Disordered” when at−2 SDs from the mean for all tests and −2.33 SDs from the mean for the AFG tests as per the manuals instructions (Keith, 2009).

**Table 2:** Name of each test, number of participants completing each test, mean z scores and percentage of classification.

**Test Number of children**	**Sub-test**	**Mean z score**	**N (%)**	**B (%)**	**D (%)**
LiSN-S 27 children	Low-cue	−0.86	56%	33%	11%
	High-cue	−1.15	44%	37%	19%
	Talker advantage	−0.94	48%	41%	11%
	Spatial advantage	−1.24	52%	26%	22%
	Total advantage	−0.77	59%	26%	15%
TEACh 27 children	Sus-AA	−0.86	63%	7%	30%
	Div-AA	−0.75	59%	19%	22%
	Sel-VA	−0.71	63%	30%	7%
	Div-AVA	−1.82	33%	15%	52%
DDT 21 children	Double–right	−1	57%	24%	19%
	Double–left	−1.1	67%	4%	29%
GiN average 18 children		−0.8	72%	11%	17%
AFG +8 dB 15 children		−1.31	53%	7%	40%
AFG 0 dB 17 children		−1.8	29%	18%	53%
FPT 11 children	Triple–right	−0.67	55%	9%	36%
	Triple–left	−0.60	45%	18%	36%

*AFG, Auditory Figure Ground; B, Borderline; D, Disordered; DDT, Dichotic Digits Test; Div-AA, Divided Auditory Attention; Div-AVA, Divided Auditory-Visual Attention; FPT, Frequency Pattern Test; GiN, Gaps-in-Noise; LiSN-S, Listening in Spatialized Noise-Sentences; N, Normal; Sel-VA, Selective Visual Attention; Sus-AA, Sustained Auditory Attention; TEACh, Test of Every Attention for Children*.

Twenty of the 27 children met criteria for APD diagnosis under the standard APD battery described previously. Pearson's partial correlation analysis was conducted on these 20 children with confirmed APD to examine the correlations between attention and AP tests in this subgroup. Scores from the right and left ear of the GiN and DDT were averaged as they were not significantly different. All assumptions of linearity, absence of outliers and normality of distribution (Shapiro-Wilk test, *p* > 0.05) were met. Gender and performance in the WNV test were controlled in the model, while age was not a covariate as all scores were converted into z scores. Results are outlined in Table 3.

**Table 3:** Pearson's partial *r* values between types of auditory/visual attention and auditory processing tests and number of children completing each test.

**Sample size**	**20**									**19**		**15**	**12**	**11**	**11**
	**Sus-AA**	**Div-AA**	**Div-AVA**	**Sel-VA**	**LiSN-S Low-cue**	**LiSN-S High-cue**	**LiSN-S Talker adv**.	**LiSN-S Spatial adv**.	**LiSN-S Total adv**.	**CCC-2 SLI**	**CCC-2 PLI**	**DDT**	**GiN**	**SCAN3 AFG 0 dB**	**SCAN3 AFG +8 dB**
Sus-AA	1.00	0.76^***^	0.51^*^	0.01	0.23	−0.01	−0.09	0.26	−0.09	0.53^*^	0.16	0.53	0.53	−0.43	−0.39
Div-AA		1.00	0.53^*^	0.27	0.25	0.08	−0.11	−0.03	0.00	0.66^**^	0.24	0.68^*^	0.27	−0.62	−0.42
Div-AVA			1.00	0.37	−0.15	−0.02	0.15	−0.21	0.05	0.21	0.01	0.76^**^	0.63	0.00	0.14
Sel-VA				1.00	0.07	0.16	−0.07	−0.28	0.15	0.24	−0.04	0.45	−0.16	−0.17	0.11
LiSN-S Low-cue					1.00	0.22	0.02	0.35	−0.15	0.30	−0.05	0.09	−0.53	0.31	−0.45
LiSN-S High-cue						1.00	0.11	0.12	0.93^***^	0.24	−0.15	−0.48	0.12	−0.31	−0.15
LiSN-S Talker adv.							1.00	0.08	0.10	−0.05	−0.22	0.39	−0.30	0.18	−0.04
LiSN-S Spatial adv.								1.00	−0.03	0.09	−0.01	−0.31	−0.12	−0.11	−0.60
LiSN-S Total adv.									1.00	0.13	−0.14	−0.49	0.34	−0.41	0.10
CCC-2 SLI										1.00	0.59^*^	0.51	0.00	−0.62	−0.34
CCC-2 PLI											1.00	0.19	0.01	−0.22	−0.03
DDT												1.00	0.04	−0.04	0.34
GiN													1.00	0.04	0.06
SCAN3 AFG 0 dB														1.00	–
SCAN3 AFG + 8 dB															1.00

*This table only includes results from the 20 children diagnosed with APD. Gender and performance in the non-verbal ability test were controlled in the model. Some children were not tested in all tests and that was due to either lack of comprehension of task or lack of time. Adv, Advantage; AFG, Auditory Figure Ground; APD, Auditory Processing Disorder; Avg., Average; CCC-2, Children's Communication Checklist−2; DDT, Dichotic Digits Test; Div-AA, Divided Auditory Attention; Div-AVA, Divided Auditory-Visual Attention; GiN, Gaps-in-Noise; LiSN-S, Listening in Spatialized Noise–Sentences; PLI, Pragmatic Language Impairment; Sel-VA, Selective Visual Attention; SLI, Specific Language Impairment; Sus-AA, Sustained Auditory Attention; TEACh, Test of Everyday Attention for Children*.

* *p < 0.05*,

** *p < 0.01*,

*** *p < 0.001*.

**Figure 1:**
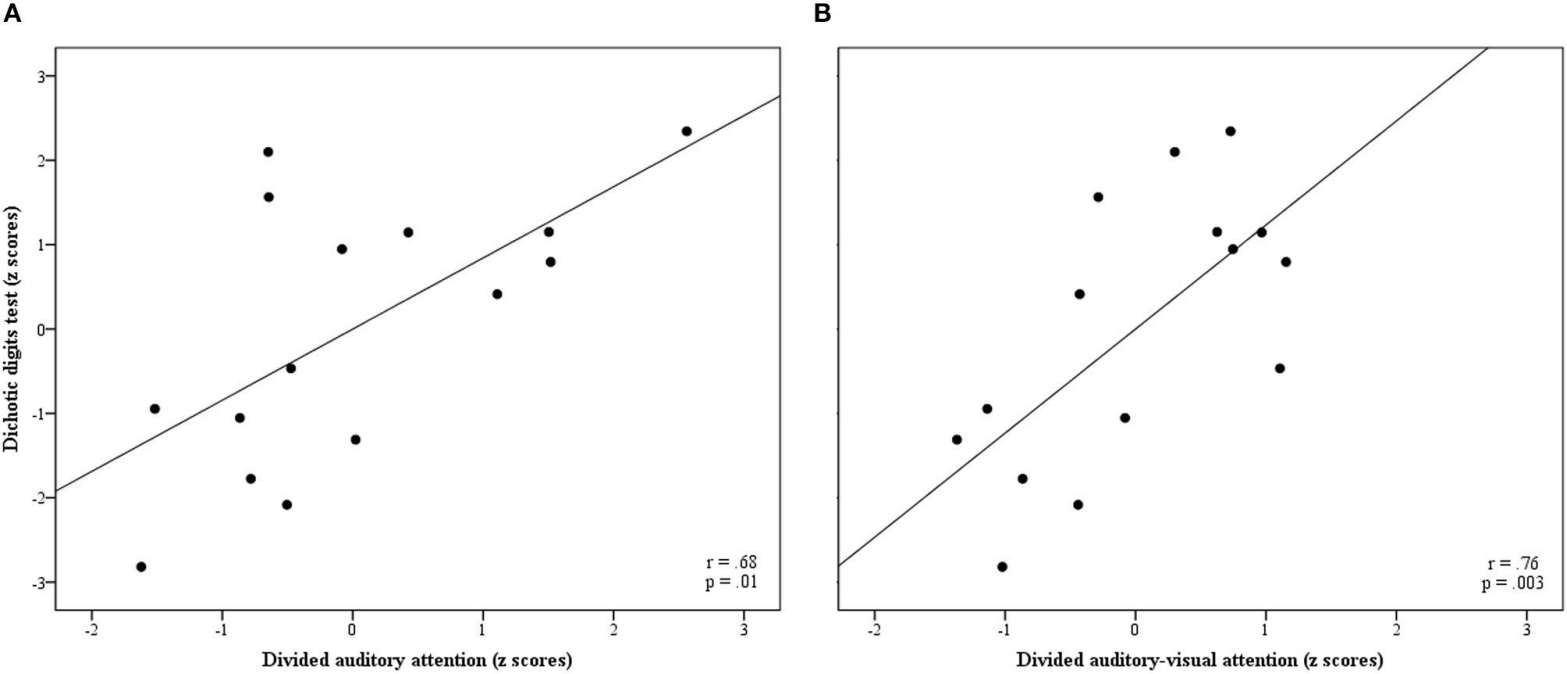
Partial regression plots **(A)** between Div-AA and DDT z scores and **(B)** between Div-AVA and DDT z scores. Pearson's partial correlation coefficients and significance values are also included in each graph. DDT, Dichotic Digits Test; Div-AA, Divided Auditory Attention; Div-AVA, Divided Auditory-Visual Attention.

There were some intercorrelations between some TEACh conditions. The TEACh Sus-AA condition correlated with the Div-AA and Div-AVA conditions, *r*_(18)_ = 0.76, *p* < 0.001 and *r*_(18)_ = 0.51, *p* = 0.03, respectively, while Div-AA correlated with Div-AVA, *r*_(18)_ = 0.53, *p* = 0.02. Divided auditory attention and Div-AVA both demonstrated strong associations with DDT scores, *r*_(13)_ = 0.68, *p* = 0.01, and *r*_(13)_ = 0.76, *p* = 0.003, respectively. These two correlations are also graphically detailed in Figure 1.

Sustained auditory attention and Div-AA both correlated with the CCC-2 SLI score, *r*_(17)_ = 0.53, *p* = 0.03, and *r*_(17)_ = 0.66, *p* = 0.004, respectively. There were also some associations between same-test conditions. Finally, LiSN-S High-cue associated with LiSN-S Total Advantage, *r*_(18)_ = 0.93, *p* < 0.001, whereas the CCC-2 SLI score correlated with the CCC-2 PLI score, *r*_(17)_ = 0.59, *p* = 0.01.

### Comparison of batteries

Table 4 shows disordered performance of each child in the tests and the classification of individual cases under the standard APD Battery and the three proposed batteries; AD, APD (without ADs) and i-APD Batteries. Diagnosis based on the CCC-2 questionnaire is also included in the table. First, a Cochran's Q test analysis was conducted to compare the proportion of children identified as disordered under the standard APD Battery, the AD Battery and the APD (without ADs) Battery. Both assumptions for sample size adequacy were met (i.e., sample size *n* ≥ 4 and sample size, *n*, multiplied by number of related groups, *k, nk* ≥ 24). The diagnostic yield between these three Batteries was significantly different, χ^2^_(2)_ = 11.200, *p* = 0.004. This was followed up by pairwise comparisons using Dunn's procedure with a Bonferroni correction for multiple comparisons with adjusted *p*-values reported. The APD Battery, with 74% diagnoses, was significantly higher than the AD Battery with 30% diagnoses, *p* = 0.003. The other comparison that was examined between the diagnostic yield of the APD Battery and the APD (without ADs) Battery (44%) was non-significant.

**Table 4:** Children's performance on each test based on SDs and identification as disordered according to the four diagnostic batteries in the study; APD, ADs, APD (without ADs) and i-APD.

**Child**	**Sus-AA**	**Div-AA**	**Div-AVA**	**Sel-VA**	**AFG +8 dB**	**AFG 0 dB**	**DDT**		**GiN**	**FPT**		**APD Battery**	**AD Battery**	**APD (without ADs) Battery**	**i-APD Battery**	**CCC-2**
							**R**	**L**		**R**	**L**					
C01									-	D	D					PLI
C02					-	D			-	D	D	D		D		ASD
C03	D		D		-	D	D	D	D	-	-	D	D			SLI
C04					-	D						D		D		
C05			D		-	D										
C06					-	D			D			D		D		
C07		D			-					D	D	D		D	D	ASD
C08	D	D	D	D		-		D		-	-	D	D			SLI
C09					D	-	-	-				D		D		
C10			D		-	-	D	D	-	D	D	D		D	D	SLI
C11	D		D			-			D			D	D			PLI
C12			D	D	D	-		D		-	-	D		D	D	SLI
C13						-				-	-	D-SPD		D		PLI
C14	D		D					D	-	-	-	D	D			SLI
C15			D						-	-	-					SLI
C16		D	D		D	-		D	-	-	-	D	D			-
C17			D		-	D						D-SPD		D	D	
C18					D	D	-	-	-	-	-	D		D		PLI
C19								D		-	-					
C20	D	D	D		-	-	D		-	-	-	D	D			
C21					D	-										SLI
C22	D	D	D			D		D		-	-	D	D			PLI
C23						D				-	-					-
C24					-		-	-		-	-	D-SPD		D		SLI
C25	D						D			-	-	D		D	D	SLI
C26	D	D	D		D	-	D			-	-	D	D			SLI
C27			D				-	-	-	-	-					PLI
Out of total												20/27	8/27	12/27	5/27	18/25
Percentage out of total (%)												74	30	44	18	72

*Out of total numbers and percentages are also presented. Black indicates performance dropping −3 SDs from the mean and dark grey −2 SDs from the mean (and −2.33 SDs for the AFG tests). For easier reading, all cases identified with a disorder under each battery were highlighted in light grey. Dash indicates incompletion of a test. AD, Attention Deficit; AFG, Auditory Figure Ground; APD, Auditory Processing Disorder; ASD, Autism Spectrum Disorder; D, Disordered; DDT, Dichotic Digits Test; Div-AA, Divided Auditory Attention; Div-AVA, Divided Auditory-Visual Attention; FPT, Frequency Pattern Test; GiN, Gaps-in-Noise; i-APD, inattentive subtype of APD; L, Left; PLI, Pragmatic Language Impairment; R, Right; Sel-VA, Selective Visual Attention; SLI, Specific Language Impairment; SPD, Spatial Processing Disorder; Sus-AA, Sustained Auditory Attention*.

Finally, as seen in Table 4, the proportion of children diagnosed under the proposed i-APD Battery was 5 out of 27, which makes up 18% of the sample.

## Discussion

### Sustained auditory attention

Previous research has shown that Sus-AA correlates with DDT (Sharma et al., 2009; Gyldenkærne et al., 2014; Tomlin et al., 2015), but their samples comprised both children suspected of and diagnosed with APD. Our correlation analysis in the group of children with confirmed APD diagnosis found a moderate but insignificant correlation between Sus-AA and DDT, *r* = 0.53, *p* = 0.06. Given the small sample size of the study and the wide confidence interval of the correlation coefficient, it is not possible to draw meaningful conclusions about the relationship between Sus-AA and DDT from the data. None of the other AP tests (GiN, LiSN-S, AFG + 8 dB, AFG 0 dB) showed significant correlations with Sus-AA, which agrees with the literature on APD-suspected children (Sharma et al., 2009; Gyldenkærne et al., 2014; Tomlin et al., 2015). Results from Table 4 reveal that 40% of children diagnosed with APD had disordered performance on the Sus-AA test (8 out of 20). Auditory Processing Disorder heterogeneity is substantiated with this result as poor Sus-AA performance is only present in a sub-group of the APD diagnosed children (40%).

### Divided attention

Divided auditory attention and Div-AVA demonstrated a strong correlation with DDT in our sample of children diagnosed with APD. Literature on APD has not yet looked at correlations between divided attention tasks and AP tests in an APD population, making the present study the first to give evidence on the relationship between these two types of attention (Div-AA and Div-AVA) and DDT in children diagnosed with APD. Divided auditory attention tasks and DDT tasks both require listening to two separate streams simultaneously and it is thus not surprising to observe such associations between their test scores. This means that during dichotic listening, divided attention might be used and/or similarly, in Div-AA tasks binaural integration skills could be employed. It could also be argued that the use of the DDT for diagnosing APD should be reconsidered, granted the relationship it has with divided attention tasks. It is not possible, though, through this analysis to determine to what extent the deficits in attention (divided and sustained) cause poorer performance in the AP measures or vice versa and how the separate deficits contribute to the problems the child has in real life. Bigger sample sized studies that would include tests that control for task attention demands and complexity of auditory test stimuli would be better suited to provide answers to these questions. These findings serve as a basis for future research on divided attention and APD.

It can be observed in Table 4 that large proportions of children with APD diagnosis face deficits in both divided attention tasks; 30% in Div-AA (6 out of 20 children) and 55% in Div-AVA (11 out of 20). These findings could point to an underlying deficit in divided attention tasks in children with APD. Pairing our findings with results from another study, which showed increased cognitive load in divided attention tasks (Mattys and Palmer, 2015), it can be argued that when children with APD are faced with such tasks poor mechanisms of allocating attentional resources (Lavie, 2005; Forster et al., 2014) are exhibited. However, Sus-AA correlated with both the divided attention tasks, while Div-AA and Div-AVA correlated, as well. The TEACh manual does not examine the correlations between its tasks, making it impossible to draw direct comparisons with our findings. However, the intercorrelations between these TEACh subtests are not surprising, since the specific Sus-AA task is part of both the Div-AA and Div-AVA tests while the literature further supports this overlap in circuit activation between different types of attention (Sarter et al., 2001). This could mean that children's deficits in divided attention tasks could be due to inherent Sus-AA deficits (or the other way around). On the other hand, it could be argued that during divided attention tasks, children might use sustained attentional resources, too. This intercorrelation between these three auditory attention tests could also explain the broad deficits in auditory attention some children face (see Table 4), as deficits in either sustained or divided attention could interact with each other and influence performance these tasks.

Finally, the lack of association between divided attention tasks and the rest of the AP tests shows that these AP tests are not strongly influenced by attention (since they also did not correlate with Sus-AA) and that they are good tools to use in an APD diagnostic battery. The DDT, revealing strong correlations with divided attention, should be reconsidered as an APD diagnostic tool, given the possible influence it might have from attention. Nevertheless, this argument needs further investigation, primarily to determine the direction of causality between these two measures. Alternatively, there is some evidence that divided attention could be linked to the function of the splenium of the corpus callosum and that children with ADHD have smaller splenium (Hutchinson et al., 2008; van der Knaap and van der Ham, 2011). Paired with the argument that corpus callosum dysfunction causes deficits in dichotic processing (Westerhausen and Hugdahl, 2008; Musiek and Weihing, 2011), it could be supported that both divided attention and DDT might require interhemispheric transfer in the auditory modality and that the latter test could still be used as an AP measure.

### Selective visual attention

Another novel finding is that AP tests in our sample did not correlate with Sel-VA. It can thus be argued that broader attention deficits (defined as deficits in both visual and auditory attention) might not be the problem with APD children. However, further investigation of this relationship and view is required, since the sample size was small and the confidence interval of the correlation coefficient wide. Besides, 10% of the APD diagnosed children had rates of disordered performance in the visual attention test. While this is comparatively lower than the rates in the three tests of auditory attention (which ranged from 30 to 55%), it still exceeds the 2% disordered threshold in the test's normative sample (Manly et al., 1999). This 10% represents 2 cases out of the 20 children with APD diagnosis. One of these two children had all the three other auditory attention TEACh tests in the disordered range, whereas the other child only had Div-AVA scores at−2 SDs from the mean. This could mean that only a small proportion of children with APD (in this sample 2 out of 20) face deficits in both visual and auditory tasks (and hence being at risk of global attention deficits), while most of the cases with failed attention scores are centred around deficits in auditory types of attention. As there are no previous studies looking at Sel-VA in children with APD, more studies need to employ different types of visual attention tests to help better characterise the nature of ADs in these children.

### LiSN-S and other measures

None of the LiSN-S conditions had significant correlations neither with the attention tests nor with the CCC-2 subscales. The development of the LiSN-S and calculation of the scores for each condition is such that minimises the effect language and communication factors have on task performance (Cameron and Dillon, 2007), thus explaining the lack of associations between LiSN-S tasks and CCC-2 subscales. The influence of attention on LiSN-S performance, though, has not been sufficiently investigated by previous research. In one study, the LiSN-S Low-cue, High-cue and Spatial advantage conditions did not correlate with a sustained attention composite score (auditory and visual) (Tomlin et al., 2015). Our results agree with and extend these previous findings, showing no associations between any of our attention measures (sustained and divided) and the LiSN-S conditions. Despite our findings, the LiSN-S Low-cue condition could be an indicator of auditory fatigue and thus of Sus-AA deficits, as it is the last condition administered during LiSN-S testing (National Acoustic Laboratories, 2010)^6^. Therefore, the lack of association between LiSN-S Low-cue scores and Sus-AA could be due to the small sample size. It has also been supported that the ability the LiSN-S derived scores (as are the Talker, Spatial and Total advantage) have on controlling for language factors (Cameron and Dillon, 2007) could extend to a control of confounding cognitive factors, as well (Tomlin et al., 2015), hence possibly explaining the lack of significant correlation between LiSN-S and attention. The other types of attention (divided and visual) were not expected to have a major influence on spatial selective listening, but selective auditory attention (not tested in our pilot) should be studied in relation to LiSN-S performance in future trials, granted the findings highlighting the contribution of selective auditory attention in listening-in-noise (Mesgarani and Chang, 2012; Zion Golumbic et al., 2013; O'Sullivan et al., 2015). Moreover, the LiSN-S High-cue condition had a strong significant correlation with the LiSN-S Total condition and this is expected, as both conditions measure the same abilities to use pitch and spatial cues to focus on the target (National Acoustic Laboratories, 2010)^6^. Finally, the lack of correlations between LiSN-S conditions and the rest of the AP tests makes the former a useful tool to use during APD assessments, especially for assessing spatial listening, as it appears to provide measurements not influenced by the other AP skills.

### CCC-2 questionnaire

The CCC-2 SLI composite score correlated with both Sus-AA and Div-AA. This composite score is used to screen for children at risk of SLI, thus it could be argued that this group of children might be facing some attention problems, specifically in sustained and divided auditory attention. At the same time, the CCC-2 PLI score did not correlate with any of the attention measures, pointing to dissociation of attention problems from pragmatic and non-standard language deficits. But as the CCC-2 SLI and PLI scores use only subscales from a parental questionnaire, future research should use additional behavioural language measures to assess SLI and PLI in children at risk of language and communication deficits, followed by examination of the relationships between these tests and types of attention.

In our study, 56% of children with communication impairments (identified using the CCC-2 questionnaire) also received APD diagnosis (14 out of 25 children; Table 4), while 71% of children with normal CCC-2 questionnaire scores were diagnosed with APD (5 out of 7). The CCC-2 is a questionnaire on communication and language abilities in children and the findings demonstrate that measurable communication impairment may be present in the majority of children with APD but not in all. Other studies have not specified proportions of disordered scores on the CCC-2 on APD/APD-suspected samples but one study compared the CCC-2 scores between an APD group, an SLI group and a typically developing group (Ferguson et al., 2011). The two clinical groups had significantly worse scores than the controls but were not significantly different between them. The two disorders could share comorbid characteristics but the important point is that a large proportion of children with APD have communication and language problems. The CCC-2 may thus contribute to the APD diagnostic evaluation by providing a measure for communication abilities (Ferguson et al., 2011), particularly for children who present to Audiology departments without any previous speech and language assessment. The CCC-2 results could thus inform management decision-making by identifying the need to involve additional professionals to audiologists in the management plan. But as the questionnaire informs of specific language and communication difficulties that may be present in different clinical entities (APD included), it cannot be used to diagnose APD.

### Diagnostic criteria for APD

Diagnosis of APD is an ongoing debate and there is yet no universal standard on diagnosing children with APD (American Speech-Language-Hearing Association, 2005b; British Society of Audiology, 2011a). Our pilot study examined the yield of the different batteries included, in order to assess a newly proposed procedure for diagnosing APD. First, the comparison of the yield between the standard APD Battery and the AD Battery revealed significant differences. This showed that more cases in the entire sample of children suspected of APD are diagnosed with APD rather than with an AD. The 8 children in the sample identified as having ADs, had at least two TEACh sub-test scores−2 SDs from the mean. These 8 children, even though also diagnosed with APD, appear to have an underlying AD, which could be influencing or explaining their AP deficits. But as there is no indication of causality, the reversed interpretation could be given, that their APD influences or worsens their attention problems. This leads to the question, should APD diagnosis exclude cases where attentional problems co-occur? Based on this argument, we proposed a battery where all APD cases were included except the 8 children identified as additionally having ADs. The proportion of these children (44%) was not significantly lower than the proportion diagnosed with the standard APD Battery. Therefore, it could be possible to use this proposed battery to identify children with only APD and no ADs, without significantly reducing the proportion of children diagnosed with an AP deficit.

These differential classifications proposed here, essentially aim at informing the management and treatment approaches suggested by clinicians. Subsequently, these 12 children with APD but no ADs, could receive management strategies already in place for children with APD. These include environmental modifications, auditory training and FM systems (American Speech-Language-Hearing Association, 2005b; British Society of Audiology, 2011b)^7^ Auditory training has been shown to improve several aspects in children with APD, such as phonological awareness, binaural listening, SiN and memory skills (Filippini et al., 2012; Sharma et al., 2012; Tawfik et al., 2015; Loo et al., 2016). The use of a personal FM system in the classroom by children with APD has been found to benefit language, phonological awareness and SiN ability (Johnston et al., 2009; Sharma et al., 2012). For the 8 cases of children identified as having ADs, along with their APD diagnosis, a more diverse management protocol could help better address their multiple needs. It is proposed that these children primarily receive management and treatment guidelines similar to the ones that ADHD children get, in combination with recommendations based on their specific AP deficits. The ADHD strategies could include behavioural therapy, classroom and teacher adaptations, special programs and perhaps medication (Wolraich et al., 2011). Children could also be followed by a psychologist who could provide them with services that would address that amodal area of difficulty. The specific AP recommendations could be centred around the AP tests in which they performed poorly. For instance, if a child performed poorly in the AFG SiN test then bottom-up auditory training and use of FM systems at school can be recommended to improve signal-to-noise ratio.

The proposed i-APD Battery aims at identifying APD children not classified as having ADs, but who have only one attention test−2 SD from the mean. Under this battery, five children (18%) were identified. While all five children would have been diagnosed as having APD in the absence of ADs (using the APD without ADs Battery), and thus not receive any recommendations for addressing attention problems, they do present with difficulties in one attention test that would have been otherwise gone undetected. Thus, the usefulness of the i-APD Battery is to identify cases of children with APD that appear to have an inattentive type of the disorder, but not broader auditory attention difficulties. Further examining the tests in which these children underperformed, it was observed that 4 out of 5 of them had deficits in a divided attention task and one of them in Sus-AA. Hence, a small subgroup of children with APD (but without general auditory attention deficits) might have a specific underlying difficulty in performing divided attention tasks. Therefore, this group of children, in addition to the standard APD management strategies mentioned above, could receive cognitive training targeting divided attention (Kerns et al., 1999).

### Limitations and future research

This was a small pilot study on a tertiary care centre population and selection bias is possible. Future studies need to examine these correlations on increased sample sizes for interpretation to be more meaningful. In addition, the study lacked a control group which makes it difficult to interpret the results due to attention influences on the tasks selected. The inclusion of differential batteries in our study accounting for attention aimed at tackling this issue, but studies with controls are required. Future research can include larger samples in combination with either typically developing controls or children without APD but with ADHD diagnosis. Moreover, direction of causality between divided attention tests and the DDT test could not be determined. Investigating this relationship further may help define management approaches that are better suited for this clinical group. Studies could examine the relationship between AP and attention tests in the diagnosed children under each of the three proposed batteries here (AD, APD without Ads, and i-APD), as this was not possible in the present study due to the reduced group sizes. Finally, as the study used the TEACh test, which lacks sub-tests that measure both modalities in the different types of attention, further examination of sustained, selective and divided attention in the auditory and visual modality should take place. This could help confirm the domain general attention problems children with APD face.

## Conclusion

The findings of the study have implications for clinical practice and APD diagnosis, as the proposed diagnostic procedure takes into account specific attentional deficits in order to classify children in better-defined categories. Incorporating attention tests in the APD diagnostic test battery adds information of value in terms of the potential clinical management of these children by identifying cases with predominantly inattentive type of APD, as well as children with general ADs. Results from the correlation analysis demonstrate significant correlations between divided attention measures and the DDT, suggesting reconsideration of the use of DDT for APD diagnosis. However, an undetermined direction of causality in this relationship stresses the need for further research to clarify this relationship. The non-correlation of the visual attention task with auditory processing tests and the fact that only two cases had Sel-VA deficits, suggests that the majority of APD children are not at risk of broader attentional problems, but instead face major deficits in auditory attentions tasks (sustained and auditory). Larger sample-sized trials including both visual and auditory attention tests of the same type are needed to cross-validate these findings and to clarify the relationship between attention and APD, which could in turn better inform choice of appropriate management strategies to address listening difficulties in real-life environments.

## Ethics statement

The study received ethics approval from the London–Bloomsbury Research Ethics Committee (REC). REC reference number: 14/LO/1509. Written and verbal explanation of the research aims was given to both parents and their participating children. They both had the opportunity to ask questions about the project. Assent forms were signed by children in the presence of their parents and parents in turn signed consent forms.

## Author contributions

GS: Conception, design and draft of the work, analysis, acquisition and interpretation of data, revising the work critically. V-MI: Contribution to the conception and design of the work, interpretation of data, revising the work critically. LE: Contribution to the design and draft of the work, revising the work critically. TS: Acquisition of data, revising the work critically. D-EB: Conception, design and draft of the work, acquisition and interpretation of data, revising the work critically. GS, V-MI, LE, TS, and D-EB: Final approval of the version to be published, agreement to be accountable for all aspects of the work in ensuring that questions related to the accuracy or integrity of any part of the work are appropriately investigated and resolved.

### Conflict of interest statement

The research was conducted in the absence of any commercial or financial relationships that could be construed as a potential conflict of interest. The authors declare that this study received funding from GN ReSound and Action Medical Research. Neither the funder nor the supporter were involved in the study design or collection, analysis, interpretation of the data or writing of the report. The reviewer, AL, and handling Editor declared their shared affiliation.

## Footnotes

^1^http://www.thebsa.org.uk/docs/docsfromold/BSA_APD_PositionPaper_31March11_FINAL.pdf.

^2^https://www.asha.org/policy/PS2005-00114/.

^3^http://www.asha.org/policy/TR2005-00043/.

^4^http://www.thebsa.org.uk/wp-content/uploads/2014/04/BSA_RP_PTA_FINAL_24Sept11_MinorAmend06Feb12.pdf.

^5^http://www.thebsa.org.uk/wp-content/uploads/2014/04/BSA_RP_Tymp_Final_21Aug13_Final.pdf.

^6^Interpreting LiSN-S Results and Recommendations for Management [Internet]. Available online at: https://capd.nal.gov.au/pdf/Interpreting%20LiSN-S%20Results.pdf (Accessed November 25, 2017).

^7^http://www.thebsa.org.uk/docs/docsfromold/BSA_APD_Management_1Aug11_FINAL_amended17Oct11.pdf.

## Abbreviations

AD: Attention Deficit
ADHD: Attention Deficit Hyperactivity Disorder
AFG: Auditory Figure Ground
AP: Auditory Processing
APD: Auditory Processing Disorder
ASD: Autism Spectrum Disorder
CCC-2: Children's Communication Checklist 2
DDT: Dichotic Digits Test
Div-AA: Divided Auditory Attention
Div-AVA: Divided Auditory-Visual Attention
FM: Frequency Modulation
FPT: Frequency Pattern Test
GiN: Gaps-in-Noise
i-APD: Inattentive subtype of APD
IQ: Intelligence Quotient
LiSN-S: Listening in Spatialized Noise–Sentences
PLI: Pragmatic Language Impairment
SD: Standard Deviation
Sel-VA: Selective Visual Attention
SiN: Speech-in-Noise
SLI: Specific Language Impairment
SPD: Spatial Processing Disorder
Sus-AA: Sustained Auditory Attention
TEACh: Test of Everyday Attention for Children
WNV: Wechsler Non-Verbal.
